# Retinal ganglion cell dysfunction in preclinical Alzheimer’s disease: an electrophysiologic biomarker signature

**DOI:** 10.1038/s41598-021-85010-1

**Published:** 2021-03-18

**Authors:** Samuel Asanad, Christian M. Felix, Michele Fantini, Michael G. Harrington, Alfredo A. Sadun, Rustum Karanjia

**Affiliations:** 1 Doheny Eye Centers-UCLA, Pasadena, CA USA; 2 Department of Ophthalmology and Visual Sciences, University of Maryland School of Medicine, Baltimore, MD USA; 3 Charles R. Drew University of Medicine and Science, Los Angeles, CA USA; 4 David Geffen School of Medicine, University of California, Los Angeles, Los Angeles, CA USA; 5 Department of Ophthalmology, Sacro Cuore Don Calabria Hospital, Negrar, Italy; 6 Huntington Medical Research Institutes, Pasadena, CA USA; 7 Department of Ophthalmology, University of Ottawa, Ottawa, ON Canada; 8 Ottawa Hospital Research Institute, Ottawa, ON Canada

**Keywords:** Diagnostic markers, Alzheimer's disease

## Abstract

The current study evaluated retinal function using electroretinography (ERG) in cognitively healthy (CH) participants with preclinical Alzheimer’s disease (AD), as classified by cerebral spinal fluid (CSF) Aβ_42_/Tau ratio. Individuals with normal retinal morphology ascertained by spectral-domain optical coherence tomography were enrolled. Full-field ERG, pattern PERG, and photopic negative response (PhNR) were performed in 29 adult participants (58 eyes). Amplitude and implicit times of the ERG wave components were analyzed. Preclinical AD participants showed marked retinal ganglion cell dysfunction relative to controls. The PhNR was significantly diminished in preclinical AD relative to controls. PhNR amplitude and N95 implicit time differentiated CH individuals with CSF biomarkers of AD pathology with 87% sensitivity and 82% specificity. These quantitative electrophysiologic findings expand our understanding of early retinal functional changes that precede cognitive decline in AD. Retinal ganglion cell dysfunction, as detected by ERG, may be a clinically useful, non-invasive in vivo biomarker for early disease detection, which is necessary for ultimately pursuing early intervention.

## Introduction

Alzheimer’s disease (AD), a chronic neurodegenerative disorder, is the most common form of dementia, affects over 26 million people worldwide, and is the most expensive disease in the United States at a total projected cost of $305 billion in 2020^1,2^. In addition to progressive memory loss and cognitive decline^3^, ophthalmologic impairments in contrast sensitivity, color discrimination, and motion perception have also been reported in AD^4^. Intriguingly, however, these symptoms of visual dysfunction in AD cannot be explained by cortical deficits alone and have been associated with the degeneration of anterior visual pathways, namely the optic nerve and the retina^5–10^. These ophthalmologic manifestations have not only been documented in the early stages of dementia, but even prior to presenting as a clear diagnosis of AD^8–11^.

Our laboratory provided the first histopathological evidence of optic neuropathy in AD after observing diffuse retinal ganglion cell (RGC) and axonal atrophy in postmortem optic nerve tissues derived from severe AD patients^12^. Pathologic hallmarks typical for AD in the brain have also been shown in the retina. We previously demonstrated Aβ accumulation inside and around melanopsin-immunoreactive RGCs (mRGCs) in postmortem AD eyes with corresponding degeneration of the retinal nerve fiber (RNFL) and ganglion cell layers (RGCL)^8,13–18^. These postmortem findings have been verified in vivo following the advent of optical coherence tomography (OCT). Live human studies have revealed significant retinal thinning in AD patients as well as in early-stage disease^6,19–22^. Nevertheless, the clinical utility of the retina as a biomarker for early AD remains limited^23–28^.

Pre-symptomatic AD describes cognitively healthy (CH) individuals having AD pathology many years prior to symptom onset. The combined ratio of Aß_42_ and Tau in the cerebrospinal fluid (CSF) has been shown to predict AD neuropathology with higher accuracy relative to the performance of these proteins individually, suggesting that both amyloid and Tau likely contribute, independently, to disease pathogenesis^29^. In parallel with these findings, more recent studies have also shown that the earliest preclinical AD can also be detected with greater sensitivity and specificity by the Aβ_42_/Tau ratio relative to the CSF concentrations of these biomarkers independently^30^. Notably, a recent prospective study conducted by our group demonstrated significant retinal thinning in preclinical AD participants who were biochemically confirmed for abnormal CSF Aβ_42_/Tau^31^. In addition to structural abnormalities, various AD studies have also shown functional changes in the retina using electroretinography (ERG)^32–36^. The present study evaluates, for the first time, retinal function using ERG in preclinical AD participants with structurally normal OCTs and investigates whether retinal electrophysiologic data can help discriminate between CH individuals with normal versus pathological Aß_42_/Tau ratio.

## Methods

### Human participants

The Institutional Review Boards of both the Huntington Medical Research Institutes (HMRI), Pasadena and the University of California, Los Angeles (UCLA) approved the protocol and consent forms for this study, which was conducted and performed in compliance with the ethical standards set out in the Declaration of Helsinki. Prior to enrollment, all study participants gave written, informed consent. Clinical and CSF studies were performed at HMRI, and ophthalmology assessments were conducted at the Doheny Eye Centers, UCLA, Division of Neuro-ophthalmology, Pasadena, CA.

Participants over 60 years of age were recruited locally at HMRI for aging research if they had no cognitive impairment after medical and neuropsychological assessment, using the Uniform Data Set-3 criteria of the National Alzheimer’s Coordinating Center (https://www.alz.washington.edu/WEB/npsych_means.html) and after consensus clinical conference. CSF Aß_42_/Tau ratios were determined from lumbar fluid as described^37^. MSD assay (catalog no. K15199G, MSD, Rockville, MD) was used to determine CSF levels of Aβ_42_. MSD assay (catalog no. K15121G, MSD, Rockville, MD) was used to determine CSF levels of total tau. We used a logistic regression cutoff for the CSF Aß_42_/Tau ratio that correctly classified more than 85% of probable AD patients to identify two separate cohorts of the CH participants: those with Normal Aß_42_/Tau ratio (“CH-NAT”), or those with Pathological Aß_42_/Tau ratio (“CH-PAT”). We thus defined that CH-PAT individuals as preclinical AD.

All subjects had a complete ophthalmic examination including assessment of best-corrected visual acuity (BCVA), intraocular pressure (IOP), slit lamp examination of the anterior segment, and a dilated fundus examination. Subjects were excluded from this study based on the following exclusion criteria: BCVA < 20/50; refractive error >  ± 5 diopters, spherical equivalent; poor OCT image quality defined by an image signal less than 6 due to severe cataracts or unstable fixation; IOP > 20 mm Hg; pre-existing retinal pathologies such as retinal vascular occlusion or retinal dystrophy; pre-existing ocular diseases such as glaucoma, optic neuropathy or uveitis; previous intraocular surgery or laser treatment except for cataract surgery performed at least 12 months prior to enrollment; penetrating ocular trauma; current active smoking status; and history or evidence of other neurological or psychiatric disorders, diabetes mellitus, poorly controlled systemic arterial hypertension defined by systolic > 150 and diastolic > 90, cardiovascular diseases, renal failure, substance abuse in the past 5 years, systemic corticosteroid use exceeding 6 months, and systemic autoimmune conditions with associated optic neuropathies.

### Retinal imaging

Retinal nerve fiber layer (RNFL), retinal ganglion cell-inner plexiform layer (RGC-IPL), and full macular thicknesses were measured for all participants using spectral-domain OCT (Cirrus SD-OCT, software v 6.0; Carl Zeiss Meditec). Scans were all acquired using the protocols for Optic Disc Cube 200 × 200 and the Macular Cube 512 × 128 in both eyes with pupil dilation. Following proper seating and alignment of each individual, the iris was brought into view using the mouse-driven alignment system, and the ophthalmoscopic image was focused. To acquire the Optic Disc Cube, the optic nerve head was centered on the live image, and centering and enhancement were optimized. After launching the scanning process, the instrument’s 840 nm wavelength laser beam generated a cube of data measuring 6 mm × 6 mm after scanning a series of 200 B-scans with 200 A-scans per B-scan (40,000 points) in 1.5 s (27,000 A-scans/sec). Cirrus SD-OCT algorithms were used to find the optic disc with automatic placement of a calculation circle measuring 3.46 mm in diameter symmetrically around it. Layer seeking algorithms were used to find the RNFL inner (anterior) boundary and the RNFL outer (posterior) boundary for the entire cube except the optic disc. The system extracted 256 A-scan samples from the data cube along the path of the calculation circle.

With respect to acquiring the Macular Cube, participants fixated on the central target. The ganglion cell analysis algorithm detected and measured the thickness of the macular RGC-IPL within a 14.13-mm^2^ elliptical annulus area centered on the fovea. The ganglion cell analysis algorithm processed data from 3-dimensional volume scans and measured the thickness of the macular RGC-IPL. Retinal thickness maps of the macular region were acquired using the macular cube scan within a 6 × 6-mm^2^ circular area centered on the fovea. Measurements were averaged over 9-retinal subfields, as defined by the Early Treatment Diabetic Retinopathy Study. A published, detailed description of this algorithm and how it operates is available for reference^37^. The built-in SD-OCT eye-tracking system provided reproducible measurements with a coefficient of variation of 0.5%^38^. An experienced operator captured all images. Individual scan volumes were reviewed for segmentation errors. Scans with significant motion artifacts, segmentation errors, or signal strength values less than 6 were excluded from analysis. To maximize the reflective signal, polarization was optimized and the scan with the best centration of the optic disc was consistently selected.

### Pattern electroretinogram

Transient PERG was performed to allow clear separation and evaluation of the P50 and N95 components. Examination was conducted according to the International Society for Clinical Electrophysiology of Vision (ISCEV) standards. ERGs were recorded with DTL-Plus (Diagnosys LLC, Lowell, MA, USA) microconductive thread electrodes with the fiber positioned at the lower cornea surface and secured on the temporal and nasal canthus after the application of topical anesthesia with proparacaine 0.5% (Anestalcon, Alcon, Fort Worth, TX, USA). Gold cup electrodes were placed on the temple for reference and central forehead (Fz) for ground.

Electrical signals were recorded using the Envoy Monocular Pattern Stimulus (Diagnosys LLC, United States) with occlusion of the contralateral eye. Recordings were obtained from both right and left eyes. The stimulus-generated black and white alternating contrast reversing bars (mean luminance, 50 cd/m^2^; spatial frequency, 0.033 cycle/deg; contrast, 99%; and temporal frequency, 1 Hz) were recorded without pupil dilation and were viewed through appropriate refractive correction to maximize retinal image quality over the full stimulus field. Participants were seated 5 cm from the screen and were instructed to fixate on a target at the center of the OLED display. The artifact rejection system was employed to filter large potential caused by extraocular movement and eye blinks. A minimum of 200 sweeps were recorded and included for analysis. The standard, transient response separates the PERG into wave components including a negative wave at about 35 ms (N35) followed by a positive wave at approximately 50 ms (P50) and a large, negative wave at around 95 ms (N95). PERG waveforms were visually inspected, and the P50-wave, N95-wave components were determined. The P50 amplitude was measured from the trough of N35 to the peak of P50. The N95 amplitude was measured from the peak of P50 to the trough of N95.

### Full-field electroretinogram

The ffERG was recorded with the same electrode montage as the PERG. Per ISCEV standards, the pupils were maximally dilated to approximately 8 mm in diameter following topical administration of 1% tropicamide and 10% phenylephrine. Stimuli were generated using the ColorDome ganzfeld associated with the Espion E3 system (Diagnosys LLC) with electrical signals being recorded simultaneously from both eyes. Participants were dark-adapted for a period of 20 min before recording the scotopic ERG responses (ISCEV standard dark-adapted 0.01 ERG, 3 ERG, 10 ERG). Following scotopic testing, participants were light-adapted for a period of 10 min before performing photopic ERG responses (ISCEV standard light-adapted 3 ERG and 30-Hz flicker). Recordings were averaged from 9 sweeps for each participant.

### Photopic negative response

The PhNR stimulus conditions were produced by ColorDome ganzfeld (Diagnosys LLC). Red (640 nm) stimulus flashes of 4 ms duration were presented at a 4-Hz rate on a blue (470 nm) rod saturating background. Red flash stimulation was presented at 1 cd·s/m^2^, while the blue background remained at 10 cd/m^2^. An Espion E3 was used to record PhNR waveforms. Six sets of 25 sweeps of 150-ms duration were recorded with bandpass filtering between 0.3 and 300 Hz at the six stimulus flash intensities. Each of the six repetitions was manually filtered to remove eye blink and other motion artifacts, and an average of the remained responses was generated for each eye. Recordings were obtained from both right and left eyes. PhNR waveforms were visually inspected, and the a-wave, b-wave, and PhNR components were determined.

### Statistical methods

Group comparison analysis of the ERG response was made using mixed model repeated measures with unstructured covariance. Group (CH-NAT, CH-PAT), ERG protocol (PERG, ffERG, PhNR), ERG parameters (P50, N95, a-wave, b-wave, PhNR, amplitude and implicit time), and eye laterality (OD, OS) were fixed effects; and age and gender were covariates.

Repeated measure multivariable logistic regression (Generalized Linear Mixed Model) based on the forward Wald stepwise procedure was used to examine the unique contributions of ERG wave-components in group classification of CH-PATs while avoiding multicollinearity. Only subjects with all data points available (all ERG protocols and all wave-components for both eyes) were included. The final model constituted independently significant predictors. Receiver operating characteristic (ROC) curve analysis was used to assess the potential diagnostic ability of the binary classification system according to the area under the curve (AUC) and to derive a cutoff for predicted event probability. Statistical significance was assumed at *p* < 0.05. Analysis was performed using SPSS V.20 package software.

## Results

Fifteen participants were classified as CH-PAT (mean age ±  standard deviation 76.5 ± 6.6 years) and 14 as CH-NAT (mean age 75.9 ± 8.5 years). There were no differences between the groups for age and gender. There were no significant group differences for RNFL, GC-IPL or macular full-thicknesses as measured by SD-OCT (Table 1).

**Table 1:** Cohort demographics, retinal and cerebrospinal fluid (CSF) data. RNFL, retinal nerve fiber layer; RGC-IPL, retinal ganglion cell-inner plexiform layer. CH-NAT, cognitively healthy with normal amyloid/tau ratio; CH-PAT, cognitively healthy with pathologic amyloid/tau ratio. Data are provided as mean ± standard deviation.

	CH-NAT	CH-PAT	P
No. of Participants (eyes)	14 (28)	15 (30)	–
Mean Age, years	75.9 ± 8.5	76.5 ± 6.6	0.6
Women (Men)	10 (4)	12 (3)	0.4
RNFL Thickness (µm)	88.1 ± 8.3	84.24 ± 7.4	0.5
RGC-IPL Thickness (µm)	74.5 ± 6.0	74.1 ± 5.8	0.8
Macular Thickness (µm)	280.6 ± 15.1	272.1 ± 14.9	0.3
CSF Aβ_42_/Tau	4.5 ± 1.3	1.3 ± 0.4	** < 0.0001**

### PERG

**Figure 1:**
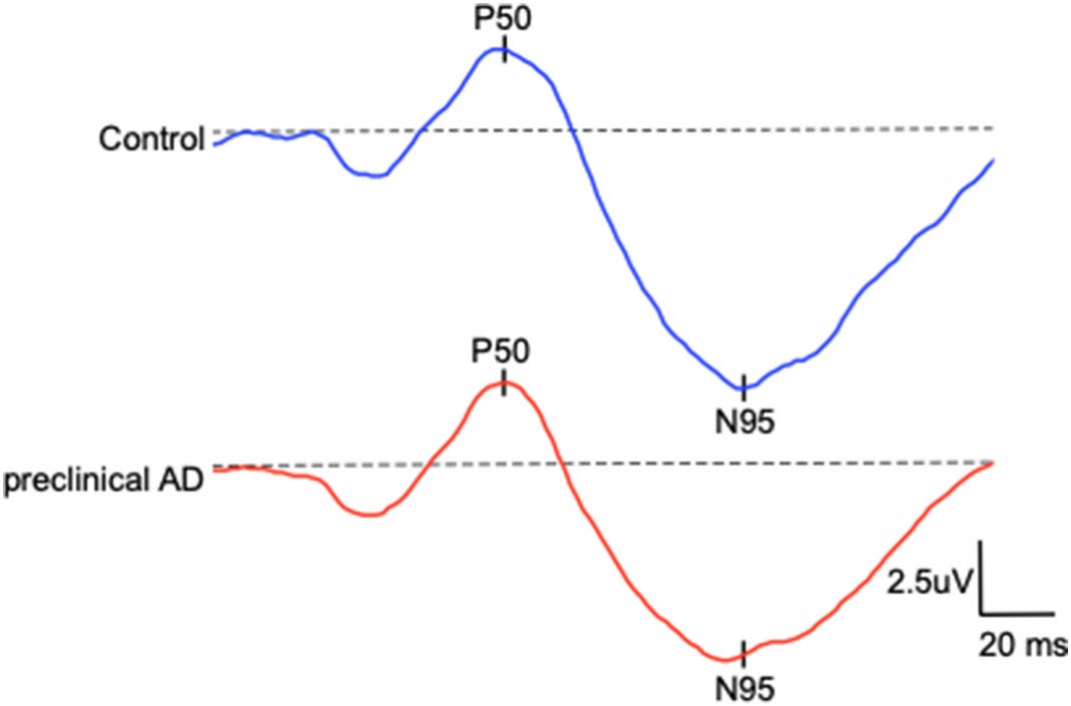
Depicts the recorded pattern electroretinogram responses in representative preclinical Alzheimer’s disease (AD) (mean N95 ± SD: 5.7 ± 2.3 µV) and control participants (mean N95 ± SD: 6.1 ± 1.7 µV).

**Figure 2:**
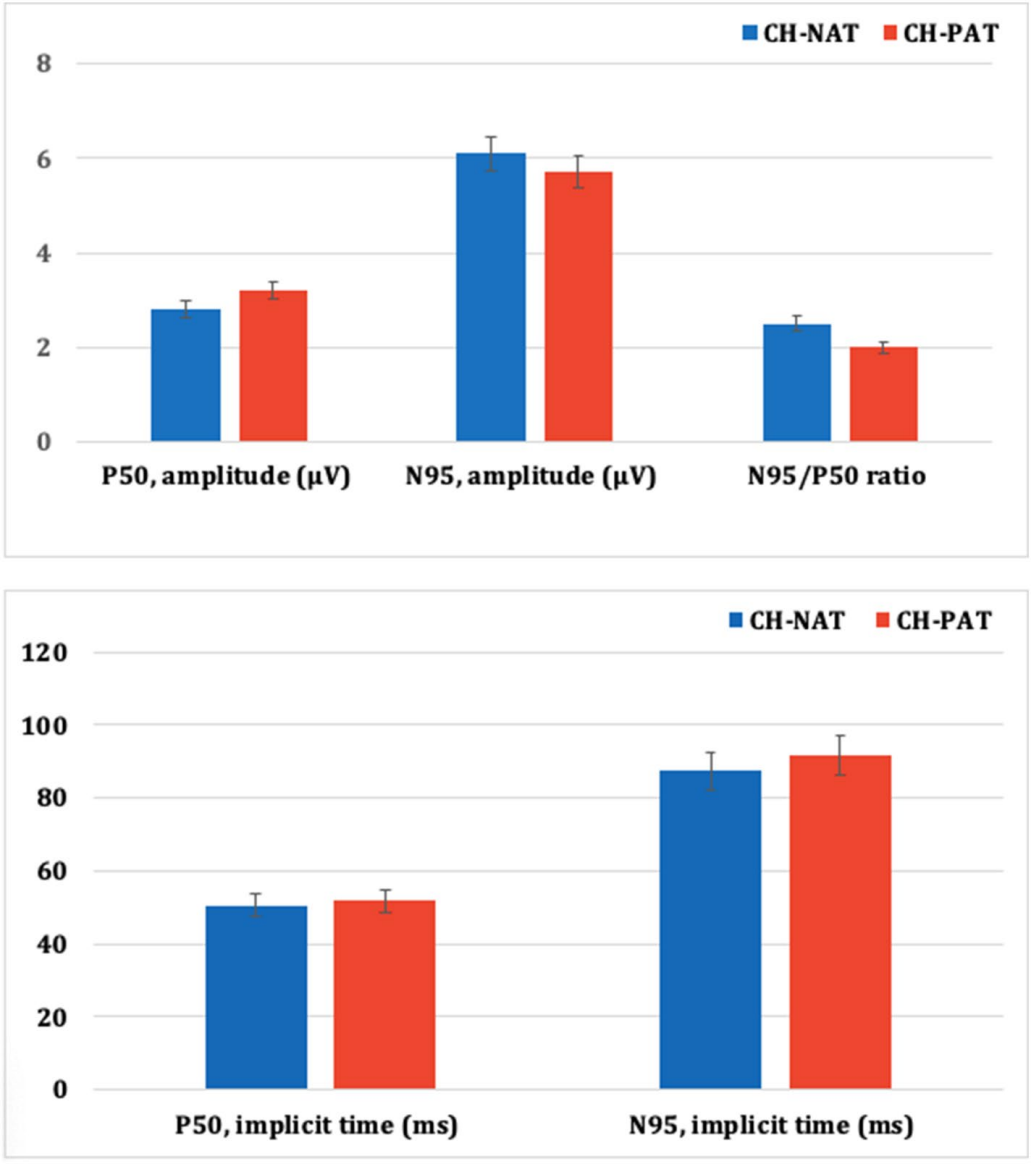
Comparison of the recorded amplitudes (top) and implicit times (bottom) pattern electroretinogram responses between CH-NAT and CH-PAT participants. CH-NAT, cognitively healthy with normal amyloid/tau ratio; CH-PAT, cognitively healthy with pathologic amyloid/tau ratio. Error bars denote standard error.

Figure 1 depicts the recorded PERG responses in representative CH-PATs and CH-NATs. As shown in Fig. 2, mean statistical analysis showed no statistically significant differences in N95, P50, or N95/P50 ratio between the two groups (*p* > 0.05 for all).

### ffERG

When using a 3 cd*s/m^2^ stimulus intensity, there was a significant delay in the a- (*p* = 0.006) and b-wave components (*p* = 0.01) of the photopic ffERG response in CH-PATs relative to CH-NATs (Table 2). There were no statistically significant differences in a- and b-wave amplitudes or implicit times for the remaining scotopic or photopic ffERG protocols between the two groups (*p* > 0.05 for all).

**Table 2:** Waveform analysis results of the full-field electroretinogram in CH-NATs and CH-PATs. Illustrates amplitude and implicit times of the dark-(DA) and light-adapted (LA) retina in response to ISCEV-defined stimulus flash intensities (cd*s/m^2^). Abbreviations: CH-NAT, cognitively healthy with normal amyloid/tau ratio; CH-PAT, cognitively healthy with pathologic amyloid/tau ratio. Data provided as mean ± standard deviation.

	CH-NAT	CH-PAT	P
DA 0.01 cd*s/m^2^ b-wave, amplitude, µV	253.9 ± 105.7	222.4 ± 87.7	0.21
DA 0.01 cd*s/m^2^ b-wave, implicit time, ms	97.2 ± 15.2	95.7 ± 13.2	0.73
DA 3 cd*s/m^2^ a-wave, amplitude, µV	179.5 ± 56.0	190.5 ± 57.4	0.6
DA 3 cd*s/m^2^ a-wave, implicit time, ms	19.8 ± 2.9	20.3 ± 3.2	0.65
DA 3 cd*s/m^2^ b-wave, amplitude, µV	327.0 ± 82.3	334.3 ± 120.1	0.96
DA 3 cd*s/m^2^ b-wave, implicit time, ms	57.2 ± 6.1	57.7 ± 10.0	0.71
DA 10 cd*s/m^2^ a-wave, amplitude, µV	229.6 ± 70.6	229.0 ± 79.3	0.91
DA 10 cd*s/m^2^ a-wave, implicit time, ms	14.8 ± 1.1	15.2 ± 1.8	0.46
DA 10 cd*s/m^2^ b-wave, amplitude, µV	133.6 ± 62.7	132.5 ± 55.8	0.67
DA 10 cd*s/m^2^ b-wave, implicit time, ms	58.5 ± 5.4	59.1 ± 5.7	0.59
LA 3 cd*s/m^2^ a-wave, amplitude, µV	29.0 ± 21.2	33.0 ± 17.1	0.47
LA 3 cd*s/m^2^ a-wave, implicit time, ms	14.1 ± 1.8	15.5 ± 1.7	**0.006**
LA 3 cd*s/m^2^ b-wave, amplitude, µV	91.3 ± 30.5	106.1 ± 37.2	0.17
LA 3 cd*s/m^2^ b-wave, implicit time, ms	31.9 ± 1.2	33.5 ± 2.7	**0.01**
LA 30 Hz flicker, amplitude, µV	74.8 ± 39.4	90.1 ± 36.0	0.19
LA 30 Hz flicker, implicit time, ms	28.7 ± 2.2	30.2 ± 2.5	0.06

### PhNR

**Figure 3:**
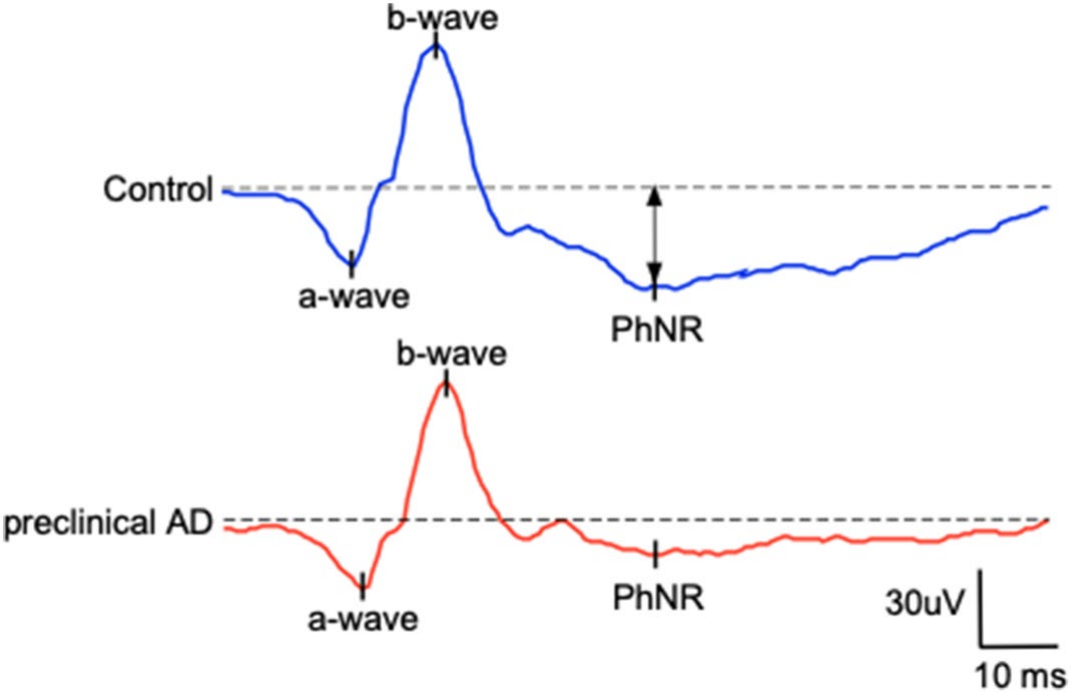
Depicts the recorded photopic negative responses (PhNR) in representative preclinical Alzheimer’s disease (AD) and control participants. As illustrated by the double-headed arrow, the PhNR was markedly diminished in preclinical AD as compared to controls.

**Figure 4:**
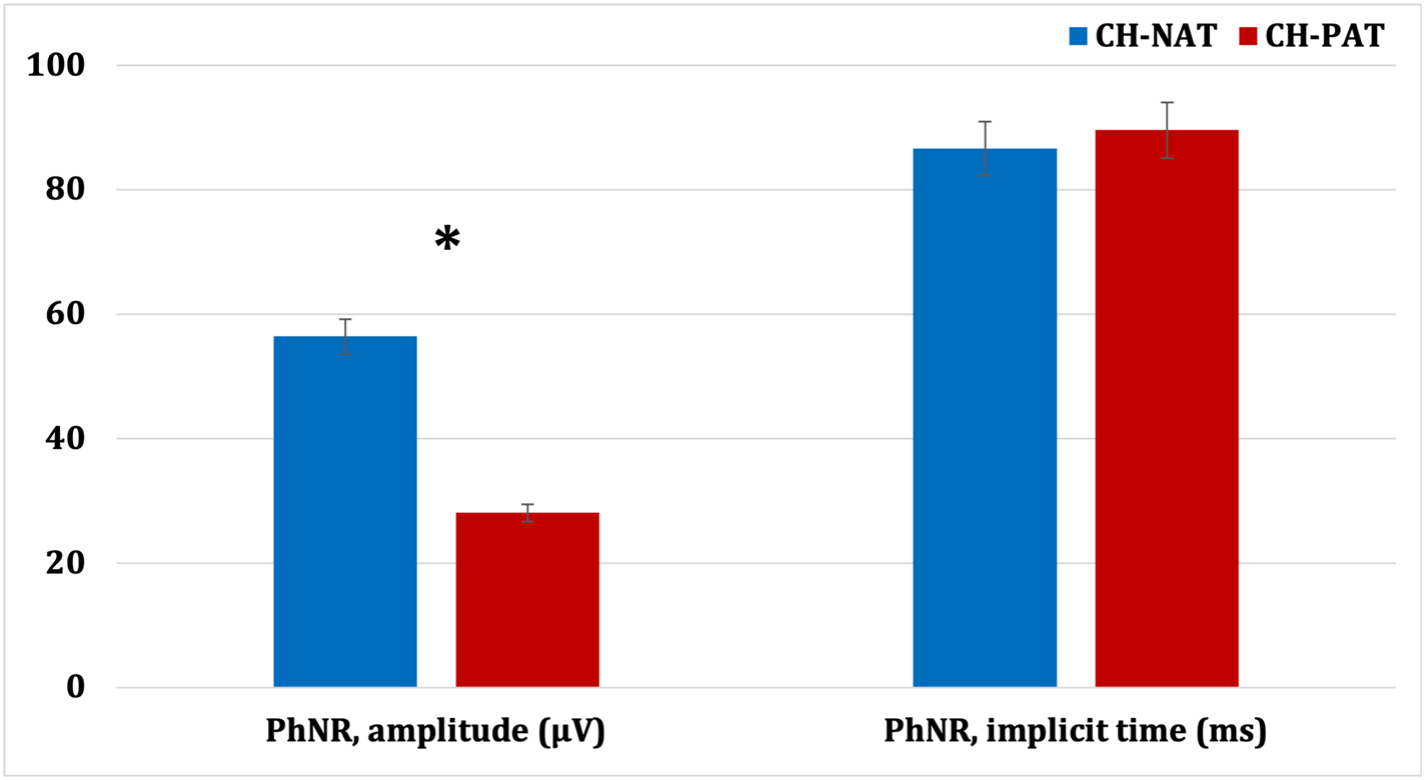
Photopic negative response (PhNR) comparison between CH-NAT and CH-PAT participants. CH-NAT, cognitively healthy with normal amyloid/tau ratio; CH-PAT, cognitively healthy with pathologic amyloid/tau ratio. Error bars denote standard error, * p < 0.05.

Figure 3 depicts the recorded PhNR responses in representative CH-PATs and CH-NATs. As shown in Fig. 4, PhNR amplitude was significantly decreased in CH-PATs relative to CH-NATs (50.1% reduction; *p* = 0.003). There was no statistically significant delay in PhNR implicit time between the two groups (*p* > 0.05).

### Predicting CH-PAT versus CH-NAT group classification

**Figure 5:**
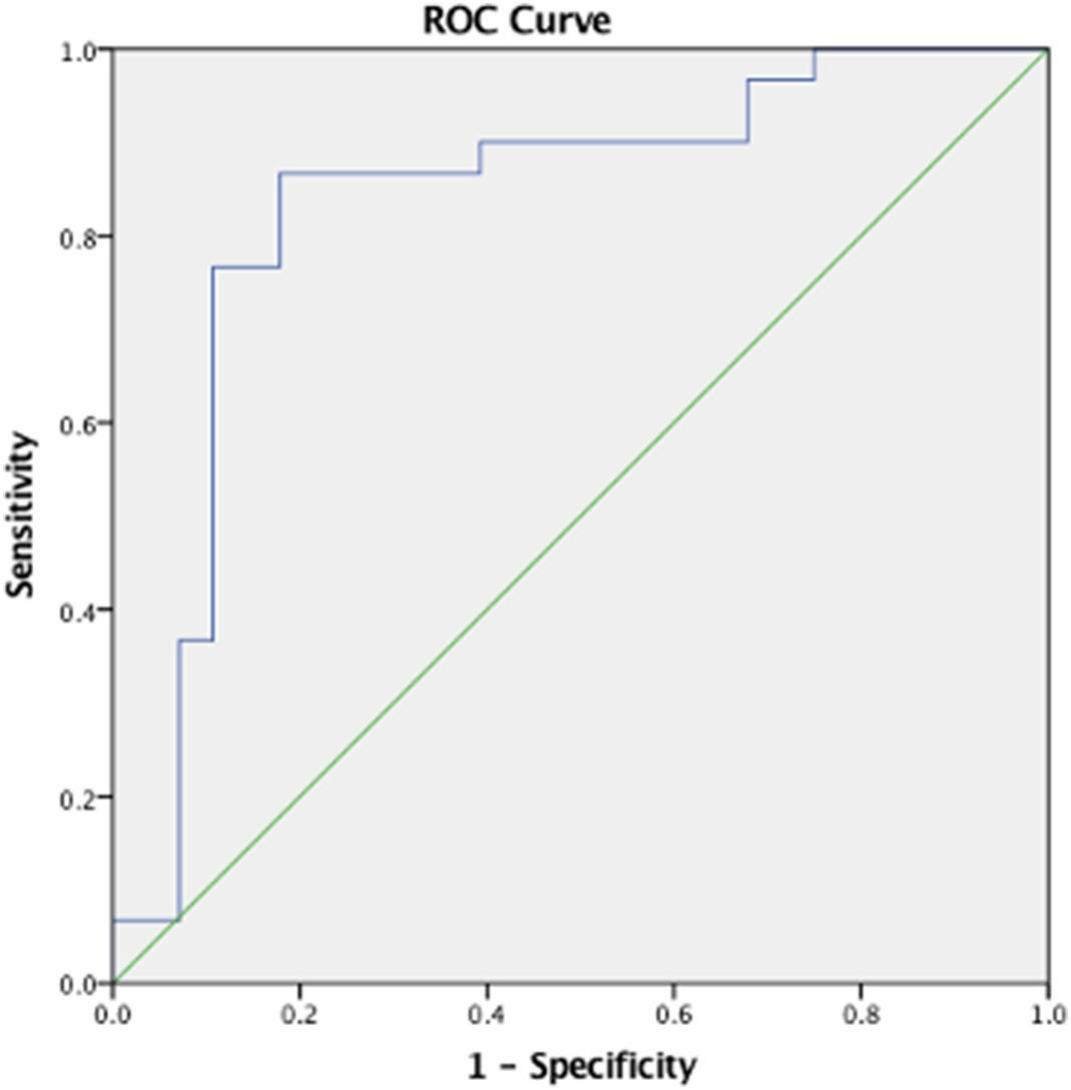
Representation of the ROC curve of the regression analysis to demonstrate which parameters of ERG predict CH-NAT versus CH-PAT group classification. The area under the curve (AUC) of PhNR amplitude and N95 implicit time was 0.84.

Logistic regression was used to predict CH-PAT group classification (dependent variable) from ERG indices (independent variables). After running the analysis for all ERG parameters (PERG, ffERG, PhNR, amplitude and implicit time), the strongest model included only PhNR amplitude and N95 implicit time as predictors (*p* < 0.0001). Upon determining the best model (PhNR amplitude and N95 implicit time), a cutoff for predicted event probability was chosen such that sensitivity was at least 85% and specificity was maximized. The selected cutoff of 0.5 yielded 87% sensitivity and 82% specificity (AUC = 0.84) (95% CI 0.72–0.95) (Fig. 5).

## Discussion

This is the first study to conduct an in vivo electrophysiologic evaluation of the retina in pre-symptomatic AD. We objectively assessed both inner and outer retinal function in human participants by PERG, ffERG, and PhNR analysis. Intriguingly, preclinical AD eyes with normal retinal morphology by OCT exhibited significant retinal dysfunction by ERG relative to controls. We further evaluated the potential diagnostic accuracy of ERG for preclinical AD. Notably, retinal electrophysiologic indices correctly predicted the biochemical class of CH-NAT versus CH-PAT in cognitively healthy, older individuals with high sensitivity and specificity. These electrophysiologic findings provide novel insights into early retinal functional changes that precede cognitive decline in AD. Retinal ganglion cell dysfunction may be clinically valuable as a quantitative, objective metric for non-invasive in vivo diagnosis of early disease, which is necessary for ultimately pursuing early intervention.

ERG detected significant RGC dysfunction in pre-symptomatic AD. Previous studies have demonstrated RGC functional deficits in symptomatic AD. Katz et al. first provided electrophysiologic evidence of RGC dysfunction in AD, as reflected by a diminished PERG response in patients relative to healthy controls^32^. Additional studies conducted by Trick et al. and Parisi et al. similarly showed an abnormal PERG response in AD^33,39^. Recent reports have also detected RGC functional loss in treatment naïve as well as in early stages of AD by PERG^35,36^. Notably, however, studies conducted by Strenn et al. failed to detect significant differences in PERG responses between AD patients and controls^40^. Discrepancies in ERG findings may be attributed to the challenges of unequivocally diagnosing AD clinically, and also the reduced cooperation typically seen with elderly and cognitively compromised patients. The current study analyzed the PhNR as a measure of inner retinal function. Notably, the PhNR was markedly diminished as compared to the PERG in preclinical AD. The PhNR has shown to be an effective measure of RGC function in optic neuropathies including optic atrophy, optic neuritis, and glaucoma^41–43^. More recently, our laboratory demonstrated the clinical utility of the PhNR in Leber’s hereditary optic neuropathy (LHON), whereby the PhNR was not only decreased in affected LHON patients, but also asymptomatic LHON carriers^44^. Compared to PERG, the PhNR is less prone to interfering factors including uncorrected refractive error or unsteady fixation, as frequently seen with older adults^43,45^. The absence of these constraints in the PhNR allows for a direct measure of RGC physiology. Our ERG findings further support the PhNR as a more robust, objective metric of RGC function and suggest its potential utility as an accessible clinical marker in older adults with pre-symptomatic AD.

Outer retinal function was relatively spared in pre-symptomatic AD. The photopic ffERG was delayed in preclinical AD when using a 3 cd*s/m^2^ stimulus intensity. Despite statistical significance, however, we only noted a ~ 1.5 ms increase in implicit time which does not suggest clinically significant outer retinal dysfunction of the cone system when compared with reference values from our normative data base and other published data sets^46^. In line with this viewpoint, we also did not observe significant differences in the ffERG photopic or scotopic responses at alternative signal intensities, which overall suggest preserved photoreceptor function in preclinical AD eyes. Our findings are consistent with prior studies in advanced AD disease stages, which have similarly reported preserved outer retinal function in AD, as measured by ffERG^32,40^. This pattern of retinal dysfunction, whereby the inner retina is selectively affected relative to the outer retina, clinically parallels histologic findings reported in AD. In a previous study conducted by our group, postmortem tissues histopathologically confirmed for Aβ deposition and derived from patients neuropathologically confirmed for severe AD exhibited marked retinal atrophy most pronounced at the inner layers involving the RGCs. These morphological changes corresponded with the distribution of retinal Aβ deposits in these tissues, as previously demonstrated by our laboratory as well as others^8,13,47–49^. These histopathologic results corroborate our retinal electrophysiologic findings and support the premise of primary and early involvement of RGCs in AD.

We further evaluated the discriminatory potential of ERG functional measures for group classification of CH-PATs versus CH-NATs. Higher significance for a given parameter does not automatically imply stronger predictivity, so we examined all ERG indices, as opposed to a priori selection of significant parameters from initial group comparison analysis^50^. Among all ERG parameters, PhNR amplitude together with N95 implicit time constituted a robust model for distinguishing the two cognitively healthy cohorts with high sensitivity and specificity. Previous studies demonstrating N95 prolongation in patients with symptomatic AD further supports the potential predictive value of this parameter^34,36^. These findings suggest that retinal electrophysiologic data have sufficient sensitivity and specificity to differentiate patients with CSF biomarkers of AD pathology.

We recently conducted a prospective study evaluating retinal structural morphology by OCT in CH-PAT and CH-NAT participants^30^. Preclinical AD individuals exhibited significant retinal thinning of the RNFL, which represents the axonal fibers connecting the ganglion cells to the brain. Notably, RNFL thickness classified CH-NAT versus CH-PAT with 87% sensitivity and 56.3% specificity^31^. The current study evaluated retinal function in premanifest AD participants with in vivo normal retinal thickness. ERG detected significant RGC dysfunction, despite normal structural morphology by OCT, and yielded a superior diagnostic performance (87% sensitivity and 82% specificity). Our findings of ERG abnormalities in the absence of in vivo retinal thinning suggests the notion that functional changes precede structural changes in AD, as indicated by previous reports^36^. Alternatively, retinal functional deficits assessed by ERG are more likely to be measured before detectable structural changes by OCT.

Limitations of this study include those associated with an exploratory approach. The statistical models await validation, and the findings should be replicated and applied to different populations to assess both neurological and ophthalmological disease, physiological, and cultural/ethnic specificity. Nevertheless, the present article represents the first electrophysiologic assessment of the retina in preclinical AD. In addition, preclinical AD and control participants were specifically classified according to Aβ_42_/Tau. This classification scheme further strengthens the significance of our findings given the superior sensitivity and specificity of Aβ_42_/Tau relative to the CSF concentration of these biomarkers alone^30^. Glaucoma is another common ocular disease that has been implicated as sharing commonalities with AD^47^. However, the relationship between these two disease entities remains controversial and additional studies are warranted to investigate their similarities. Importantly, all participants enrolled in our study underwent complete ophthalmic examination to exclude potentially confounding ocular pathologies. The current study did not analyze axial length. Retinal stretching and cell damage associated with axial elongation could lead to retinal thinning and diminished ERG amplitude. However, these effects of axial myopia are most notable in high myopes (> 6 diopters)^51^. In the present article, participants with refractive error > 5 diopters were excluded and there were no significant differences in OCT retinal thickness between cohorts. While delayed implicit time has also been observed with increasing axial length, these effects are reportedly less remarkable^51^. Given the cross-sectional nature of our investigation, long-term longitudinal follow-up studies are necessary for the proper characterization of disease natural history. In turn, we plan to use these results as baseline values for future longitudinal studies. Further research is also necessary to precisely elucidate the mechanism of retinal pathology observed in these cohorts. Additional studies investigating the relationship between electrophysiology and alternative measures of retinal function including visual field performance testing may be particularly insightful. Nevertheless, this study suggests the utility of the PhNR as a reliable, objective and sensitive metric that directly reflects RGC physiology in presymptomatic AD. In addition, retinal electrophysiologic data, namely the PhNR and N95, may allow for improved diagnostics of AD pathology before cognitive decline, as compared to OCT structural data alone. These are important outcome measure against which to evaluate the efficacy of purported treatment strategies for AD.
